# Beyond cancer treatment: dermo-aesthetic and other wellness recommendations for breast cancer patients

**DOI:** 10.1007/s12094-024-03636-9

**Published:** 2024-08-29

**Authors:** Alejandro Falcón González, María Isabel Gallegos Sancho, Encarnación González Flores, Elena Galve Calvo, Julia Ruiz Vozmediano, Paloma Domingo García, Ricardo López Martos, Elena Sánchez Rivas, Carmen María Iglesias Urraca, Ana Isabel Gómez Calvo, Amaia De Mariscal Polo, Rocío Ramos-Medina, Maria Rivero, Virginia Martínez Marín

**Affiliations:** 1 Medical Oncology Service, Hospital Universitario Virgen del Rocío, Av. Manuel Siurot, S/N, 41013 Seville, Spain; 2 Medical Oncology Service, Hospital General de Segovia, C/ Luis Erik Clavería, 40002 Segovia, Spain; 3 Medical Oncology Service, Hospital Virgen de las Nieves, Av. de las Fuerzas Armadas, 2, Beiro, 18014 Granada, Spain; 4 Medical Oncology Service, Hospital Universitario Basurto, Osakidetza, Av. Montevideo, 18, 48013 Bilbao, Spain; 5 Director of the Vodder Physiotherapy Center, Calle Ayala, 48. 1º Izq., 28001 Madrid, Spain; 6 Oral and Maxillofacial Surgery Service, Hospital Universitario Virgen del Rocío, Av. Manuel Siurot, S/N, 41013 Seville, Spain; 7 Clínica Dres. Mario León, C. Miguel de Cervantes, 22, 06800 Mérida, Spain; 8 Plastic Surgery Service, Hospital Universitario La Paz, P.º de la Castellana, 261, Fuencarral-El Pardo, 28046 Madrid, Spain; 9 Gynecology and Obstetrics Service, Hospital General de Segovia, Luis Erik Clavería Street, 40002 Segovia, Spain; 10 Dermatology Service, Basurto University Hospital, Montevideo Etorb., 18, Basurtu-Zorrotza, 48013 Bilbao, Spain; 11 Medical Department, Pfizer Oncology, Madrid, Spain; 12 Medical Oncology Service, La Paz University Hospital, P.º de la Castellana, 261, Fuencarral-El Pardo, 28046 Madrid, Spain

**Keywords:** Aesthetic medicine, BC, Integrative care, Multidisciplinary treatment, Oncology rehabilitation, Quality of life

## Abstract

Breast cancer, a prevalent malignancy among women, has various physical and psychological impacts. This comprehensive review offers an in-depth look at multidisciplinary dermo-aesthetic intervention approaches, emphasizing the balance between oncological therapies and the management of these effects. The information presented spans specialties such as aesthetic medicine, plastic surgery, dermatology, physiotherapy, nutrition, odontology, and gynecology. This review, which serves as a clinical guide, aims to establish a safe protocol for non-medical interventions involving oncologists, physicians, and specialists from various areas in patients with breast cancer focused on improving their quality of life. This work offers personalized and integrative care strategies for the eradication of cancer. However, it is still necessary for patients to consult with their oncologist before undergoing any dermo aesthetic treatment. However, it is still necessary for patients to consult with their oncologist before undergoing any dermo aesthetic treatment.

## Introduction

The treatment of breast cancer (BC), one of the most prevalent oncological diseases in women worldwide [1, 2], involves not only the management of the disease itself but also its multiple repercussions in various areas of patient health and well-being [3]. Cancer treatments, such as chemotherapy, radiation therapy, and hormone therapy, can have significant side effects on the patient’s skin, musculoskeletal system, oral health, body image, and sexual function [4]. These effects, which vary in intensity and duration, can manifest during or after treatment, further complicating the path to recovery [5].

Cancer involves not only abnormal cell proliferation but also a chronic inflammatory microenvironment and altered hormonal communication, often influenced by unhealthy lifestyles [6, 7]. It is crucial to understand how a balanced and personalized diet can be a fundamental tool for managing overweight and obesity, key risk factors in the development and prognosis of BC. These conditions increase the risk of recurrence and mortality, alter the effectiveness of treatment, and increase the probability of developing significant adverse effects. This is mainly due to the increased activity of the aromatase enzyme in adipose tissue [8, 9], which intensifies the production of endogenous estrogens, known stimulants of tumor growth [7, 10–15], and modifies the response of the body to treatment, complicating the clinical management of BC. Furthermore, treatment has to be personalized since obesity in postmenopausal women is associated with a higher risk of developing breast cancer. In contrast, obesity in premenopausal women may confer a protective effect. This change may be due to variability in estrogen levels, which are implicated in estrogen-sensitive breast cancers and increased states of higher adiposity. BC frequently involves interventions that can significantly alter patients’ body image. Plastic surgery techniques serve reconstructive purposes and contribute to patients’ aesthetic, functional, and emotional recovery [16].

In addition to the risks associated with overweight and obesity in the development and management of BC, it is necessary to know how hormone therapy and oncological treatments directly influence various dermatological and oral conditions. By modifying the hormonal microenvironment and increasing susceptibility to side effects, these treatments produce a series of skin and oral manifestations that significantly affect the patient’s quality of life. Dermatologically, patients may experience xerosis, rash, and hand-foot syndrome, requiring careful management, including corticosteroids, antihistamines, or photoprotection. Facial changes, such as hypersensitivity to the sun, redness, and spots, as well as changes in the body, affect a patient’s personal image and require specific attention not only because of physical changes but also because of their impact on the patient's self-esteem [17, 18]. Musculoskeletal ailments and lymphedema resulting from oncological treatments can also occur, leading to the development of pain and reduced functionality [19, 20].

In relation to oral health, this is also compromised in BC patients, especially those undergoing chemotherapy, as they require specialized care to prevent and treat problems such as mucositis, dysgeusia, and xerostomia [21, 22]. These individuals can also develop genitourinary syndrome of menopause (GMS), which occurs especially in patients undergoing hormonal therapy-induced menopause and can affect sexual function, such as through dyspareunia and decreased libido [23, 24].

The diversity of effects that can occur around BC patients requires a personalized and multidisciplinary treatment approach [25]. There are critical areas that must be addressed in the patient with breast cancer, such as physical exercise or psychological support [26, 27]. However, concerning dermo-aesthetic interventions, it is necessary to consider the integration of specialties such as aesthetic medicine, plastic surgery, dermatology, physiotherapy, nutrition, dentistry, and gynecology. In this way, treatments will aim to eradicate cancer, improve patients’ quality of life, promote their recovery, and improve their general well-being.

This review aims to establish a safe protocol for non-medical interventions involving oncologists, physicians, and specialists from various areas in patients with BC. Focusing on recommendations with little available literature, we seek to improve patients’ physical well-being and quality of life.

## Dermatology

**Fig. 1:**
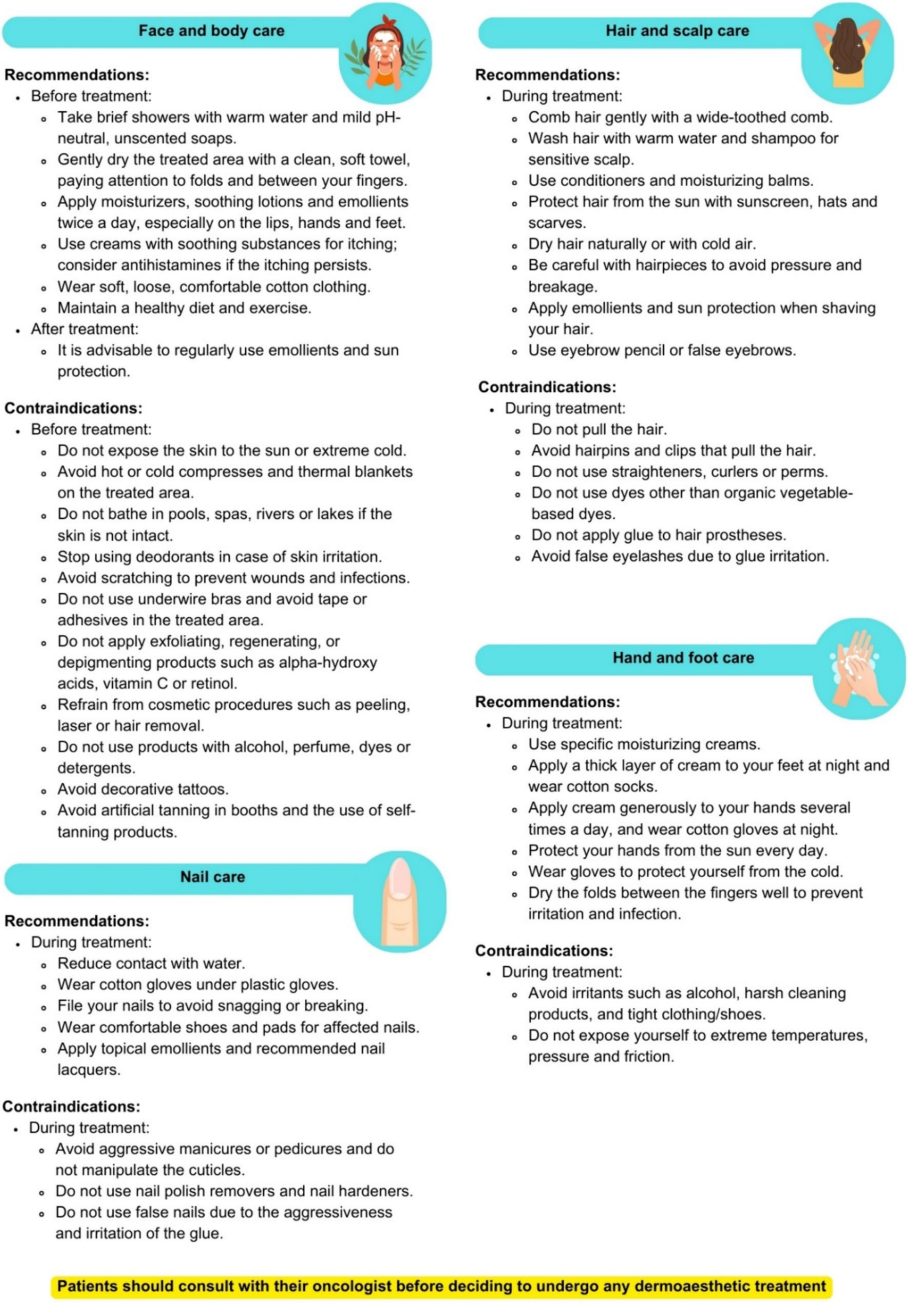
Recommendations and contraindications for dermatological care in patients with breast cancer

To minimize skin toxicity and its manifestations, facial and body care, scalp and hair treatments, hand and foot care, and nail care are recommended. The recommendations are shown in Fig. 1.

### Facial and body care

Skin changes, such as dryness, pruritus, and irritation, are common side effects during BC treatment [17, 28, 29]. Educating patients about skincare routines, including using emollients and photoprotection, and avoiding irritants are essential. Skincare formulations should be safe, additive- and fragrance-free, and easy to apply [30]. Dermatological care focuses on preparing the skin before and during treatment to minimize side effects, restore the skin barrier and restore the normal condition of the skin after treatment [3].

### Scalp and hair care

The scalp becomes more sensitive during oncological therapies, and the hair may become fragile and brittle. Approximately 65% of patients receiving chemotherapy experience alopecia, although this is usually reversible, and hair begins to recover approximately three months after stopping treatment. Most patients opt for camouflage methods, such as hair prostheses or scarves, since only 1 to 4% suffer from permanent alopecia, which requires evaluation and treatment by a dermatologist [31]. To prevent alopecia induced by chemotherapy, a scalp cooling system can be used, which reduces the risk of hair loss by half by causing local vasoconstriction and reducing the metabolic needs of hair follicles during chemotherapy sessions [30].

### Hand and foot care

Palmo-plantar syndrome is a relatively common toxicoderma that generates redness, swelling, and pain in the palms of the hands and soles of the feet. It dramatically impacts the patient's quality of life and may require a reduction in the treatment dose or even suspension of treatment [17, 28, 32].

### Nail care

Nail alterations during oncological treatments are common, although they are generally well tolerated and usually disappear when treatment is stopped. The most common symptoms are melanonychia, leukonychia, onycholysis, onychomadesis and onychorrhexis [28]. Paronychia and periungual granulomas, although generally not severe, can be debilitating for patients; therefore, strategies must be implemented to address these adverse effects [32].

## Aesthetic medicine and plastic surgery

**Fig. 2:**
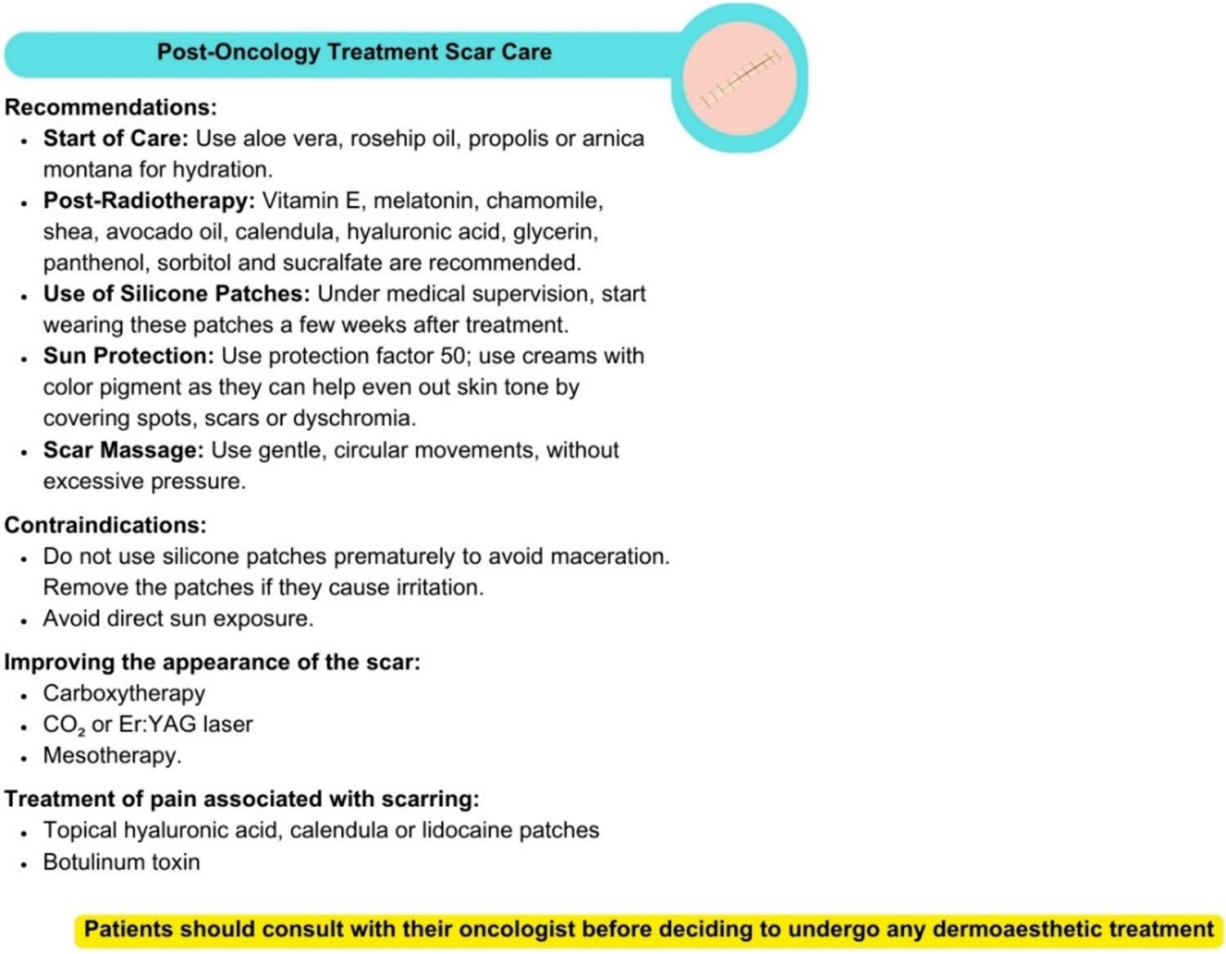
Recommendations for the care of scars after oncological treatment

In the treatment of BC, aesthetic medicine and plastic surgery play key roles, providing a comprehensive approach that ranges from preparing the skin before treatment to mitigating side effects such as radiodermatitis and dryness during treatment. Although it is recommended to wait 6–12 months after treatment for more intense procedures, the lack of a clear scientific consensus on the optimal timing for plastic surgery underscores the importance of coordination between oncology and aesthetic medicine teams [36]. These practices seek to improve the patient's physical appearance and self-image by reconstructing affected areas, treating scars, and supporting their emotional recovery and quality of life [34]. Recommendations for the care of scars after oncological treatment are shown in Fig. 2.

### Medical-aesthetic treatments

**Fig. 3:**
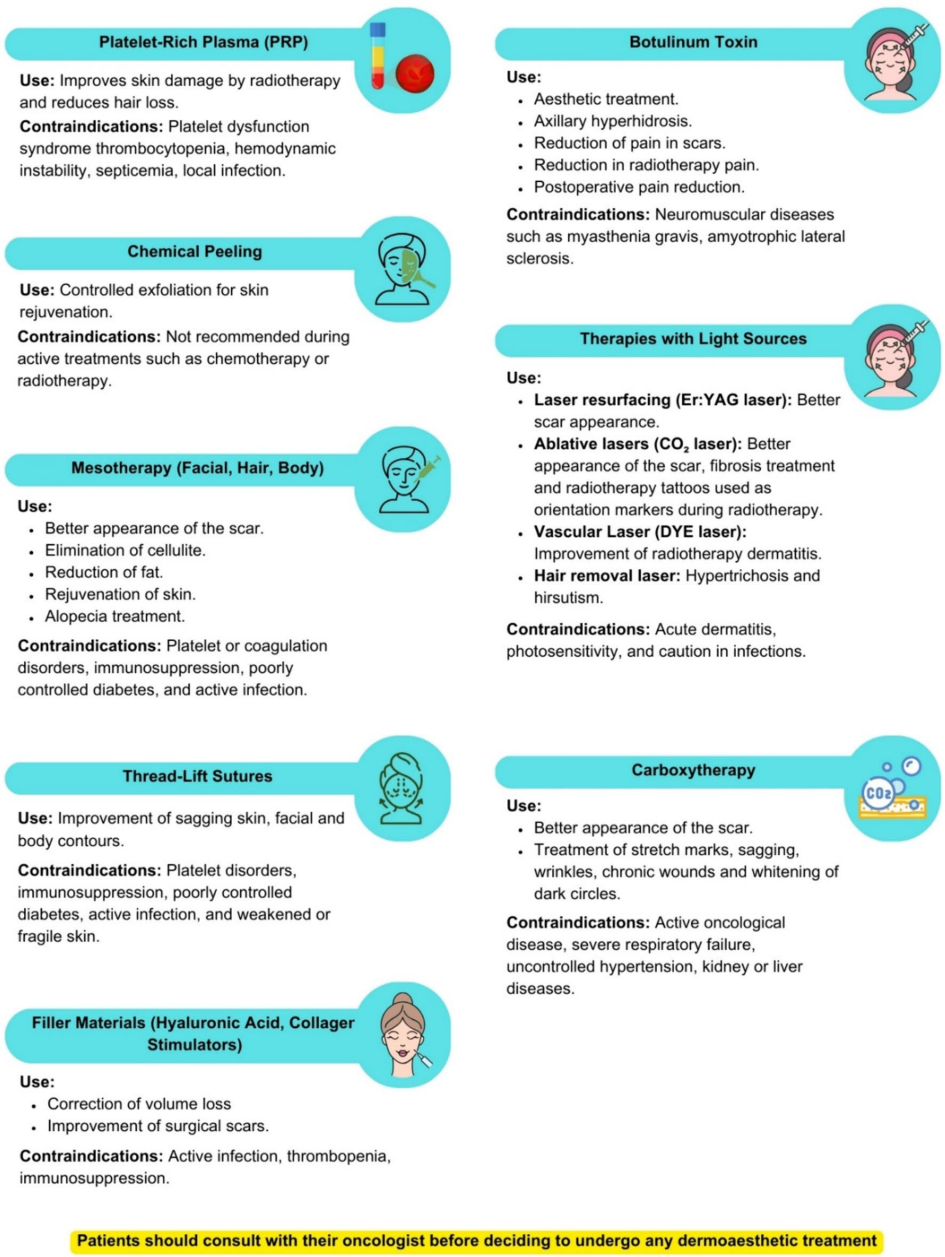
Recommendations and contraindications for medical-aesthetic treatments in patients with breast cancer

The recommendations and contraindications for medical-aesthetic treatments are shown in Fig. 3.

#### Platelet-rich plasma (PRP)

A derivative of the patient’s blood, enriched in platelets, is used to rejuvenate the skin. This treatment stimulates collagen and elastin production through growth factors that promote fibroblast proliferation, vascularization, and tissue regeneration. However, caution is advised in patients who have used nonsteroidal anti-inflammatory drugs or systemic corticosteroids in the 15 days prior to sample collection, as well as in patients with low hemoglobin (less than 10 g/dl), a blood count basal platelet count less than 150,000/μl, patients with a poor venous network, patients with neutropenia, or patients who are being treated with medications that can cause neutropenia or thrombopenia [35, 36]. Table 1 shows a comprehensive overview of the risks associated with various chemotherapy and targeted therapy agents, particularly focusing on severe toxicities such as grade 3 and 4 neutropenia and thrombopenia.

**Table 1:** Toxicity according to drug type

Toxicity	Drugs	G3 and G4 risk percentage
Plateletopenia ≥ G3 < 50,000 platelets	Chemotherapy	
	Anthracyclines Taxanes Eribulin Vinorelbine Cisplatin/Carboplatin Gemcitabine Capecitabine	≤ 5% ≥ G3 1–7% ≥ G3 1% ≥ G3 1% ≥ G3 25–30%/25–62% any degree 1–4% ≥ G3 1–3% ≥ G3
	ADC	
	Sacitunumab govitecan	1–3% ≥ G3
	AntiHER2	
	Trastuzumab emtansine Trastuzumab deruxtecan TKI (Lapatinib, tucatinib, neratinib) PARP inhibitor (Olaparib/talazoparib) Cyclin inhibitor (Palbociclib/ribociclib/abemaciclib) mTOR inhibitor (Everolimus) pi3K inhibitor (Alpelisib)	6–15% ≥ G3 3–12% ≥ G3 Infrequent 1–3%/4–11% ≥ G3 1–2%/1%/2% ≥ G3 1–3% ≥ G3 1% ≥ G3
Neutropenia and febrile neutropenia ≥ G3 < 1000 neutrophils	Chemotherapy	10–20% risk of global febrile neutropenia
	Anthracyclines Taxanes Eribulin Vinorelbine Cisplatin/Carboplatin Gemcitabine Capecitabine	11–67% ≥ G3 14–75% ≥ G3 12–57% ≥ G3 29–69% ≥ G3 25–30%/16–67% ≥ G3 any degree 6–19% ≥ G3 1–3% ≥ G3
	ADC	
	Sacitunumab govitecan	32–53% ≥ G3
	AntiHER2	
	Trastuzumab emtansine Trastuzumab deruxtecan TKI (Lapatinib, tucatinib, neratinib) PARP inhibitor (Olaparib/talazoparib) Cyclin inhibitor (Palbociclib/ribociclib/abemaciclib) mTOR inhibitor (Everolimus) pi3K inhibitor (Alpelisib)	7–8% ≥ G3 12–51% ≥ G3 Infrequent 4–6%/3–18% ≥ G3 10–56%/7–55%/5–22% ≥ G3 1–9% ≥ G3
Hemorrhage and/or risk of bleeding	AntiVEGF (Bevacizumab)	G3-4 2–5.8% G1-2 30%
Osteonecrosis mandibular	Bisphosphonates (Alendronic or Zolendronic acid) RANK inhibitors (Denosumab) Alpelisib	1–10% any degree 2–5% any degree 4% any degree

Drug	Type of toxicity	Risk of grade 3 and 4 toxicity (%)
Chemotherapy agents		
Anthracyclines	Plateletopenia, Neutropenia	≤ 5%, 11–67%
Taxanes	Plateletopenia, Neutropenia	1–7%, 14–75%
Eribulin	Plateletopenia, Neutropenia	1%, 12–57%
Vinorelbine	Plateletopenia, Neutropenia	1%, 29–69%
Cisplatin/Carboplatin	Plateletopenia, Neutropenia	25–30%/25–62% any degree, 25–30%/16–67% any degree
Gemcitabine	Plateletopenia, Neutropenia	1–4%, 6–19%
Capecitabine	Plateletopenia, Neutropenia	1–3%, 1–3%
Antibody–drug conjugates (ADC) and AntiHER2 agents		
Sacituzumab Govitecan	Plateletopenia, Neutropenia	1–3%, 32–53%
Trastuzumab Emtansine	Plateletopenia, Neutropenia	6–15%, 7–8%
Trastuzumab Deruxtecan	Plateletopenia, Neutropenia	3–12%, 12–51%
Targeted therapy agents (TKI, PARP, Cyclin, mTOR, pi3K Inhibitors)		
Lapatinib, Tucatinib, Neratinib	Plateletopenia, Neutropenia	Infrequent, Infrequent
Olaparib, Talazoparib	Plateletopenia, Neutropenia	1–3%/4–11%, 4–6%/3–18%
Palbociclib, Ribociclib, Abemaciclib	Plateletopenia, Neutropenia	1–2%/1%/2%, 10–56%/7–55%/5–22%
Everolimus	Plateletopenia, Neutropenia	1–3%, 1–9%
Alpelisib	Plateletopenia, Neutropenia	1%, Infrequent
Additional toxicity concerns		
Bevacizumab (AntiVEGF)	Hemorrhage and/or Risk of Bleeding	2–5.8% G3-4, 30% G1-2
Alendronic Acid, Zoledronic Acid, Denosumab	Osteonecrosis Mandibular	1–10% any degree, 2–5% any degree, 4% any degree

AntiHER2: Treatments directed against human epidermal growth factor receptor 2; AntiVEGF: Anti-Vascular Endothelial Growth Factor; DC: Antibody–Drug Conjugate; G3: Degree of toxicity 3; G4: Degree of toxicity 4; RANK inhibitors: Receptor Activator of Nuclear factor Kappa-B Inhibitors

#### Chemical peel

Substances with exfoliating capacity can be applied to achieve controlled peeling of the epidermis or superficial dermis and improve skin aging, texture, and tone, reduce spots and scars, and revitalize the general appearance of the skin that may be affected by side effects such as xerosis or post-inflammatory hyperpigmentation resulting from chemotherapy or radiotherapy [37]. There are many options for chemical peels, but as a general rule, they should not be prescribed to patients undergoing active treatment, given the constant alteration of barrier function associated with chemotherapy, radiotherapy, immunotherapy, and hormone therapy.

#### Facial, hair, and body mesotherapy

Mesotherapy involves vitamins, minerals, amino acids, and hyaluronic acid microinjections. Although it is generally safe and applicable before and after oncological treatments, it can yield side effects such as pain or bruising. Rarely, mesotherapy may cause serious complications, including systemic reactions. Moreover, it is crucial to consider interactions with other drugs in cancer patients [37, 38].

#### Tensioning wires

Tensioning wires consist of very fine threads, often made of biocompatible and biodegradable materials such as polylactic acid or polydioxanone, that are inserted under the skin to lift and firm sagging or wrinkled areas [39]. This treatment may particularly benefit patients with BC who experience changes in physical appearance due to oncological treatments. Initially, this treatment is a feasible option after oncological treatment, as long as there are no contraindications, such as platelet or coagulation disorders, immunosuppression, poorly controlled diabetes, or active infection. Furthermore, the placement of tension wires is not indicated in patients with weakened skin.

#### Filler materials: hyaluronic acid and collagen formation stimulators

Injections of filler materials, such as autologous fat or hyaluronic acid, are used to improve aesthetics. However, there may be side effects such as inflammation, redness, edema, pain, bruising, and, rarely, hypersensitivity reactions and granulomas. There are also risks of infection, reactivation of herpes simplex virus, and complications such as cellulitis and abscesses. Moreover, it is essential to consider immunosuppression and interactions with other treatments and to take aseptic precautions. Some materials may interfere with radiological interpretations, such as calcium hydroxyapatite in computed tomography (CT), positron emission tomography using fluorodeoxyglucose (PET-FDG), and magnetic resonance imaging (MRI) images [40].

In addition, it is crucial to monitor for the appearance of the “bolus effect” and the “callback effect,” where the skin reacts to the sun as if it were undergoing radiation therapy [41]. Attention must also be given to patients receiving anticoagulants [42], patients receiving treatments that affect collagen synthesis or wound healing, and patients receiving immunotherapy where granulomatous reactions may occur [40]. Table 2 lists the medications commonly associated with modifying wound healing and collagen synthesis processes. It categorizes these agents based on their class and mechanism of action.

**Table 2:** Drugs that reduce scarring

Medication	Class
Apixaban	Factor Xa inhibitor
Acetylsalicylic acid	NSAIDs
Azathioprine	Immunosuppressive antimetabolite
Capecitabine	Nukeloside metabolic inhibitor with antineoplastic activity
Celecoxib	Selective COX-2 inhibitor/NSAID
Clopidrogel	Platelet antiaggregant
Corticosteroids	Immunosuppressants
Cyclosporin	immunosuppressant
Dabigatran	Direct thrombin inhibitor/anticoagulant
Dipyridamole	Platelet antiaggregant
Ibuprobene	NSAIDs
Methotrexate	Antimetabolite
Mycophenolate	immunosuppressant
Naproxen	NSAIDs
Rivaroxaban	Factor Xa inhibitor/anticoagulant
Valdecoxib	Selective COX-2 inhibitor
Warfarin	Vitamin K antagonist/anticoagulant

Medication	Class	Primary action
Apixaban	Factor Xa inhibitor	Reduces thrombosis, aiding in scar reduction
Acetylsalicylic acid	NSAIDs	Anti-inflammatory, promotes healing
Azathioprine	Immunosuppressive antimetabolite	Reduces immune response, lowering scar formation
Capecitabine	Nukeloside metabolic inhibitor with antineoplastic activity	Targets rapidly dividing cells, reducing tissue disruption
Celecoxib	Selective COX-2 inhibitor/NSAID	Reduces inflammation specifically at the site
Clopidrogel	Platelet antiaggregant	Reduces platelet aggregation
Corticosteroids	Immunosuppressants	Potently reduces inflammation and immune response
Cyclosporin	immunosuppressant	Lowers immune activity to mitigate excessive scar formation
Dabigatran	Direct thrombin inhibitor/anticoagulant	Prevents thrombin formation, reduces risk of clot-induced scarring
Dipyridamole	Platelet antiaggregant	Lowers platelet aggregation, beneficial for healing without excessive scarring
Ibuprobene	NSAIDs	Anti-inflammatory properties aid in less traumatic healing
Methotrexate	Antimetabolite	Inhibits cell proliferation, useful in controlled healing to avoid scar tissue buildup
Mycophenolate	immunosuppressant	Suppresses immune responses, promoting smoother healing
Naproxen	NSAIDs	Helps reduce inflammation, which can lead to reduced scarring
Rivaroxaban	Factor Xa inhibitor/anticoagulant	Anticoagulant that reduces clotting, lowering scar tissue formation
Valdecoxib	Selective COX-2 inhibitor	Targets inflammation precisely without extensive side effects, promoting better healing
Warfarin	Vitamin K antagonist/anticoagulant	Reduces blood clotting, which can help in reducing scar formation during healing

*COX-2 inhibitor* cyclooxygenase-2 inhibitor, *NSAIDs* Nonsteroidal anti-inflammatory drugs

#### Botulinum toxin

Botulinum toxin (BTX) induces muscle paralysis by inhibiting the release of acetylcholine. BTX also inhibits nociceptive fibers, contributes to reducing pain [43–48] both after radiotherapy and after surgery, and improves the aesthetics of retractile and hypertrophic scars due to its effect on fibroblasts [49, 50]. The treatment effects have a durability of 3 to 6 months [45, 46].

In some patients, the therapeutic efficacy may be limited due to resistance or antibody formation, especially in the case of immunotherapy treatments, because they induce the activation of B lymphocytes and plasma cells against their epitopes, with the consequent production of antibodies that can cross-react with the proteins expressed by BTX. Although BTX is associated with a low frequency of side effects and is usually well tolerated by cancer patients [45], it can i cause redness, edema, hematoma, pain, and, rarely, more severe complications. BTX should be used with caution in cases of coagulation disorders and cancer patients treated with immunotherapy due to the risk of cross-antibody formation [40].

#### Therapies with light sources: pulsed light, laser, fractional radiofrequency

Lasers in oncology are mainly used to improve dermatological and aesthetic conditions related to cancer treatment. Vascular lasers can be used to treat vascular lesions, depigmentants can be used to correct pigmentation alterations, depilatories can be used to manage abnormal hair growth, and rejuvenators can improve skin quality and appearance affected by oncological therapies. In particular, erbium with neodymium (Er:YAG) laser resurfacing and ablative lasers, such as CO_2_ lasers, effectively improve the appearance of scars, treat fibrosis, and remove radiotherapy tattoos. Furthermore, the DYE vascular laser contributes significantly to improving radiotherapy-induced dermatitis. The implementation of these treatments must always be carefully evaluated and supervised by specialists, ensuring their suitability and safety for each cancer patient. Laser therapy is contraindicated in patients with acute dermatitis and photosensitivity. Caution should also be taken in treating infections [40, 51].

#### Carboxytherapy

Carboxytherapy involves the infiltration of CO_2_ through fine needles, which stimulates the repair and dilation of blood vessels, improving cellular oxygenation and circulation. This process releases catecholamines, histamines, and serotonin, promoting vasodilation and lipolytic effects against cellulite and localized fat. The goal is to promote vasodilation of the capillaries in the deepest layer of the skin. In this way, by increasing irrigation in cases where there are scars, the rearrangement of collagen fibers and, therefore, the aesthetic outcome can be improved. Additionally, it may be useful in reducing fibrosis after surgery or radiation [52]. This treatment is contraindicated in patients with active oncological disease.

### Aesthetic cabin treatments (nonmedical)

**Fig. 4:**
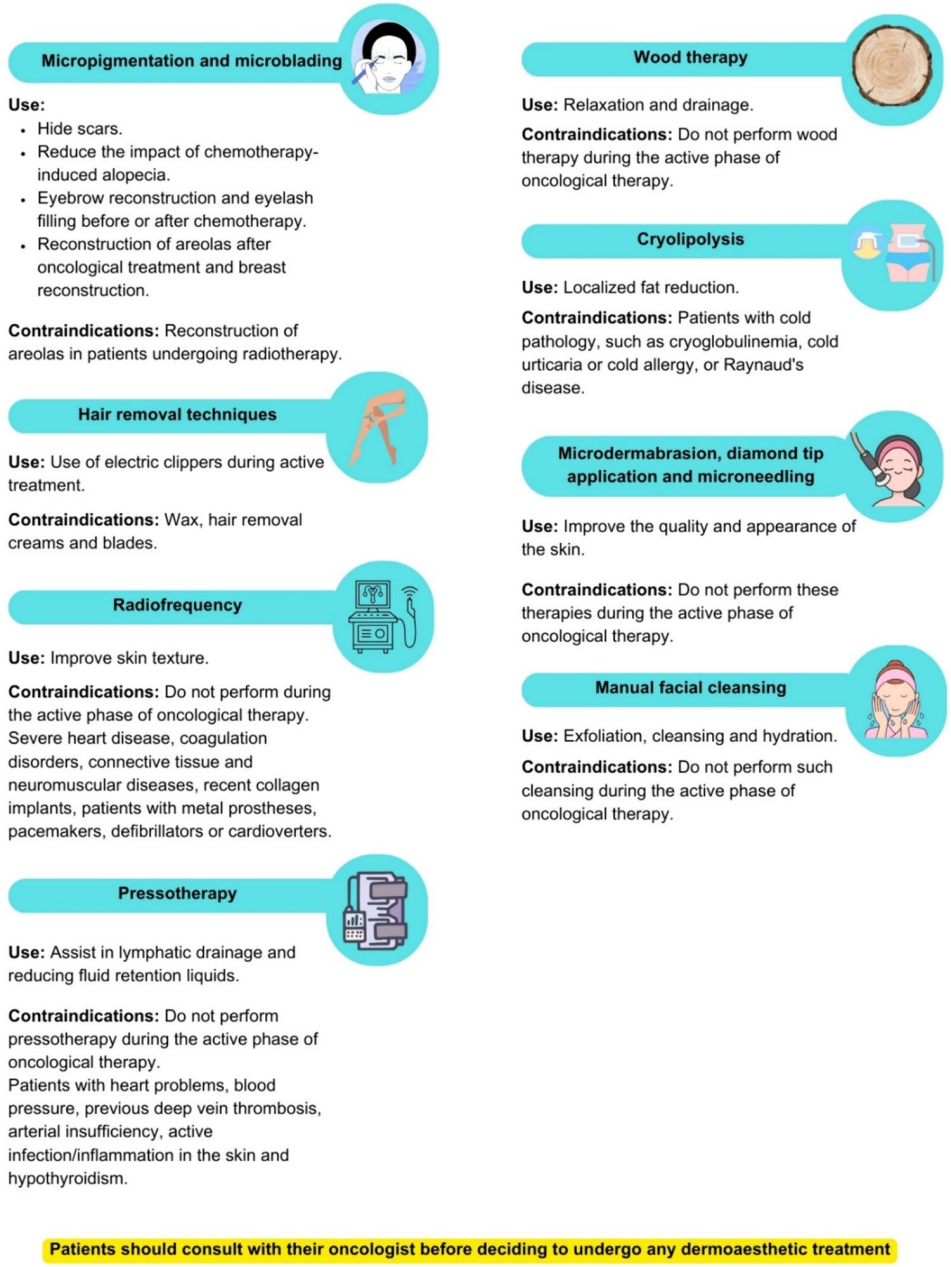
The recommendations and contraindications for aesthetic cabin treatments in patients with breast cancer

The recommendations and contraindications for aesthetic cabin treatments are shown in Fig. 4.

#### Micropigmentation and microblading

Micropigmentation and microblading are techniques by which pigments can be inserted into the skin to improve or balance aesthetic features, such as lips, eyebrows, and eye contours. These pigments fade after several months. Microblading, by making cuts, opens another door to infection; therefore, if the patient needs these techniques during immunosuppressive treatments, micropigmentation is preferable [53–55]. According to the medical criteria, performing these techniques before or after chemotherapy is recommended. It is advisable for patients to visit centers specializing in oncoesthetics [56, 57].

#### Hair removal techniques

For the removal of unwanted hair, which may arise as a side effect of certain hormonal therapies in BC treatment, the use of an electric clipper is suggested during active treatment. Afterward, medical laser hair removal may be considered. The use of wax, depilatory creams, and razors is discouraged during active treatment due to the risk of cuts and skin reactions [58].

#### Radiofrequency therapy, pressotherapy, and wood therapy

Radiofrequency uses electromagnetic waves to heat the dermis, pressotherapy applies pressure for lymphatic drainage using a pneumatic suit, and wood therapy uses wooden massage tools. These treatments are generally not recommended during active cancer therapy. Radiofrequency is contraindicated in patients with severe heart disease and other specific disorders. Pressotherapy is not suitable for treating active cancer disease, heart problems, or other conditions. Maderotherapy follows the same precautions as “oncology massage” in cancer patients (more information in section about Physiotherapy) [59].

#### Cryolipolysis

Cryolipolysis is a noninvasive technique that cools subcutaneous adipose tissue to reduce fat through cell death (apoptosis) of adipocytes. Some patients treated for BC may experience weight gain after their treatment due to hormonal changes, decreased physical activity, metabolic alterations, and emotional factors. Although cryolipolysis could be used to reduce weight gain, it is contraindicatedin patients with cold pathologies, such as cryoglobulinemia, Raynaud's syndrome or allergies. In addition, cryolipolysis has been shown to be effective in treating lipodystrophy induced by BC treatments [60, 61].There are no specific safety studies on cancer patients, and caution should be exercised due to the risk of hematomas, especially considering the platelet level and coagulation status of the patient [62, 63].

#### Microdermabrasion, diamond tip application, and microneedling

Microdermabrasion, diamond tip, and microneedling techniques can be used after cancer treatment to improve the appearance of the skin, including scars and texture. However, these techniques are contraindicated during active cancer treatment due to the risk of deep exfoliation that can compromise the skin barrier, increasing the risk of infections, delayed healing and pigmentation problems [37].

#### Manual facial cleansing or the use of noninvasive automatic systems

Facial cleansing, manual or automatic systems, includes exfoliation, cleansing, and hydration. These procedures are designed to adapt to each patient's specific needs, seeking benefits without attacking the skin. However, it is contraindicated during cancer treatment and should only be performed after these treatments with medical approval [64].

### Conventional techniques in plastic surgery

There are various breast reconstruction techniques for women who have undergone a total or radical mastectomy. Among the most common is reconstruction with the DIEP (Deep Inferior Epigastric Perforator) flap, which uses skin and fat from the abdomen without sacrificing the muscle [65], the use of breast prostheses [66], which can be placed directly or after expansion of the tissue using an expander, or the latissimus dorsi flap technique, which uses muscle, skin and fat from the back. In this section, other less-used techniques will be addressed [67].

#### Volumetric repositioning surgeries

##### Facelift or cervicofacial surgery (rhytidectomy)

The cervicofacial lift is a surgery to rejuvenate the face, reducing sagging and folds in the cheeks and jaw. It includes tissue repositioning, skin tightening, and removal of excess skin. It is ideal for improving the appearance of the cheeks and neck. It is not suitable for superficial wrinkles or sun damage. The risks included bleeding, infection, and reactions to anesthesia. Complications are rare but may include bruising, scarring, nerve injuries, and hair loss at incision sites [68, 69].

##### Upper lip lift surgery

Upper lip lift surgery is minimally invasive and seeks to shorten the distance between the nose and the upper lip, permanently increasing its volume. A cut is made below the base of the nose, leaving an almost imperceptible scar. The procedure offers immediate, natural, and permanent results [70].

##### Blepharoplasty lift surgery

Blepharoplasty is a surgery to repair sagging eyelids by removing excess skin, muscle, and fat. With age, the eyelids stretch, and the muscles weaken, causing sagging and bags. Blepharoplasty can improve vision and rejuvenate the appearance of the eyes. The results are often long-lasting, but additional surgery may be needed. The side effects include infection, bleeding, dry eyes, eyelid problems, and vision changes. It is essential to protect the eyelids from the sun after surgery [71].

#### Lipotransfer and fat volumization surgeries

##### Facial lipofilling

The facial fat transfer technique is minimally invasive, using the patient's fat to rejuvenate the face and reduce wrinkles. Facial lipofilling offers several benefits: it eliminates the risk of rejection, provides permanent results without reabsorption of fat cells, leaves almost imperceptible scars, and requires a simple postoperative period with local anesthesia. The procedure rejuvenates without affecting facial expression [72, 73].

##### NANOFAT

The nanofat technique involves harvesting stem cells from fat to create nanofat, which is then infiltrated into the skin with needles or needling devices. It is used to improve skin quality and treat dark circles and hair loss but not as a filler [74].

#### Volumetric reduction techniques

##### Liposuction

Liposuction is a surgical procedure that removes fat from specific areas, such as the abdomen or thighs, to shape the body. Postoperative inflammation decreases within weeks, and the results stabilize within months. It is suitable for people with localized excess fat but stable weight. The suspension of certain medications and prior analysis may be necessary. Risks include bleeding, anesthetic reactions, skin irregularities, fluid buildup, numbness, infections, internal puncture of organs, fat embolism, and kidney or heart problems. It is not a weight loss method [75].

##### Abdominoplasty

Abdominoplasty removes excess fat and skin from the abdomen and can tighten the abdominal muscles. The surgery, performed under general anesthesia, includes incisions to remove the skin and readjust the muscles and skin, keeping the navel in place. This approach leads to body image improvement, especially after childbirth, significant weight changes, or previous abdominal surgeries. It can be combined with liposuction and requires prior medical evaluation [76].

##### Mastopexy

Mastopexy involves lifting the breasts, removing excess skin and reshaping the breast tissue. Indicated for sagging breasts or stretched areolas, mastopexy improves the appearance and comfort of clothing. There may be changes in breast size and shape, and the areola may shrink. The scars fade over time but do not disappear completely [77].

### New techniques in plastic surgery with technology

Going through the stress of the disease and the side effects of medications leads to a tired, aged appearance of the face with secondary flaccidity and, in the body, flaccidity secondary to weight gain or flaccidity due to massive weight loss. For this, tissue repositioning surgeries with increased or decreased adipose tissue are recommended.

#### BodyTite liposuction

BodyTite is a minimally invasive body and facial contouring treatment using radio frequency-assisted liposuction (RF-assisted liposuction). This approach provides fast, scar-free 3D results in a single session. It is performed under local anesthesia or sedation and allows for almost immediate recovery. FaceTite focuses on the face and small areas, and AccuTite focuses on precise areas. There are few complications, such as pain and bruising. It is ideal for eliminating localized fat and flaccidity in various areas. Post-treatment, the use of compression girdles with specific regimens is recommended. Facial compression is not recommended, and sports should be limited to the first ten days post-intervention [78].

## Odontology

Oncological treatments have significant effects on oral health. Tamoxifen, an antiestrogenic drug, can cause depression and fatigue, affecting the ability to maintain good oral hygiene [22, 79, 80]. Aromatase inhibitors, which lower estrogen levels more than tamoxifen, can impair periodontal health, reduce saliva flow, increase the risk of gingivitis and cavities, and alter taste. On the other hand, bisphosphonates and antiresorptives, which are used to prevent bone loss, can lead to osteonecrosis of the jaw (MRONJ) [22, 79, 80].

**Fig. 5:**
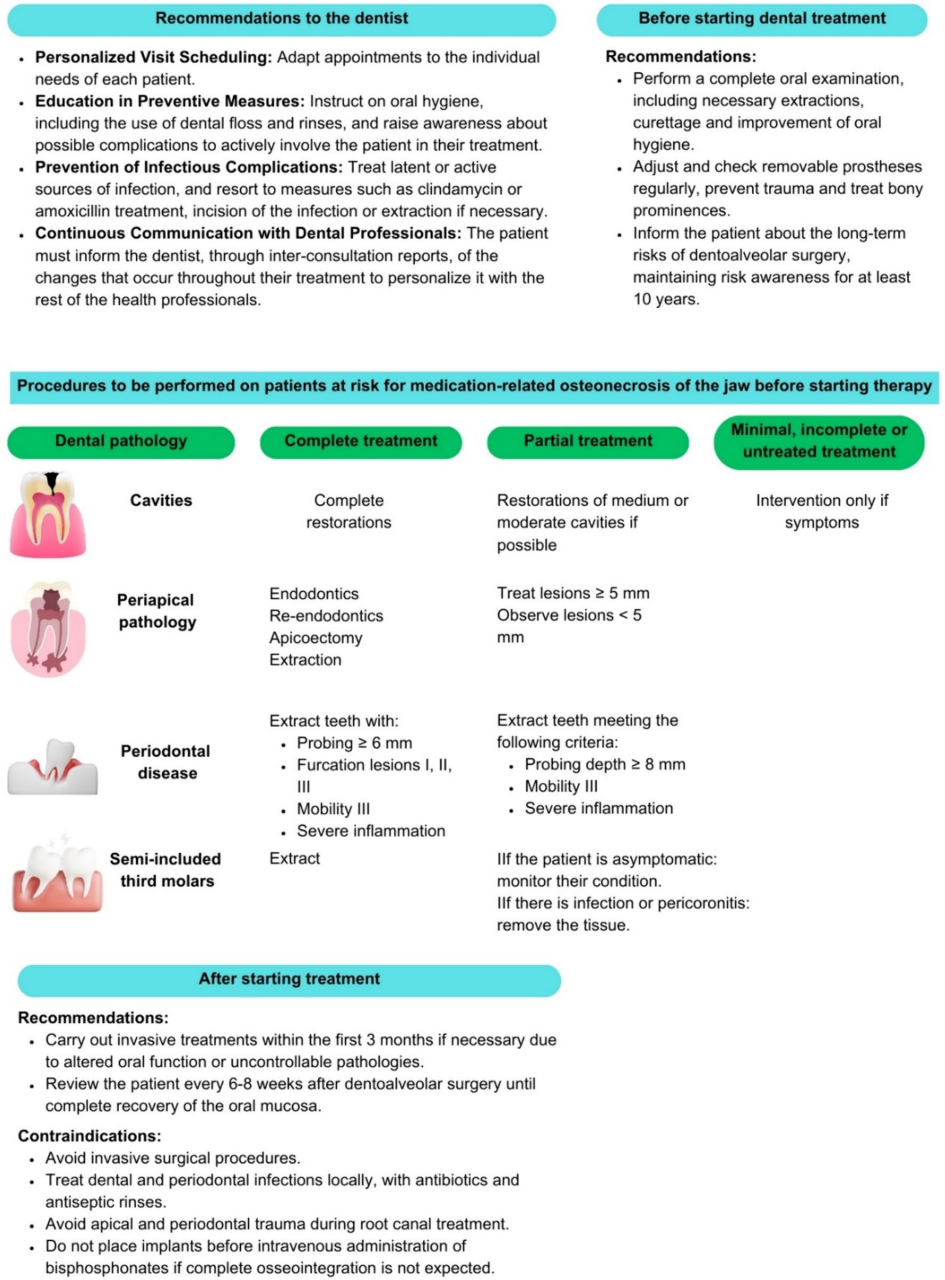
Recommendations and contraindications for dental treatments in patients with breast cancer

Oral health professionals should be informed about the effects of oncological drugs on oral health and possible complications, as well as the recommendations to consider before and during oncological treatment (Fig. 5).

**Fig. 6:**
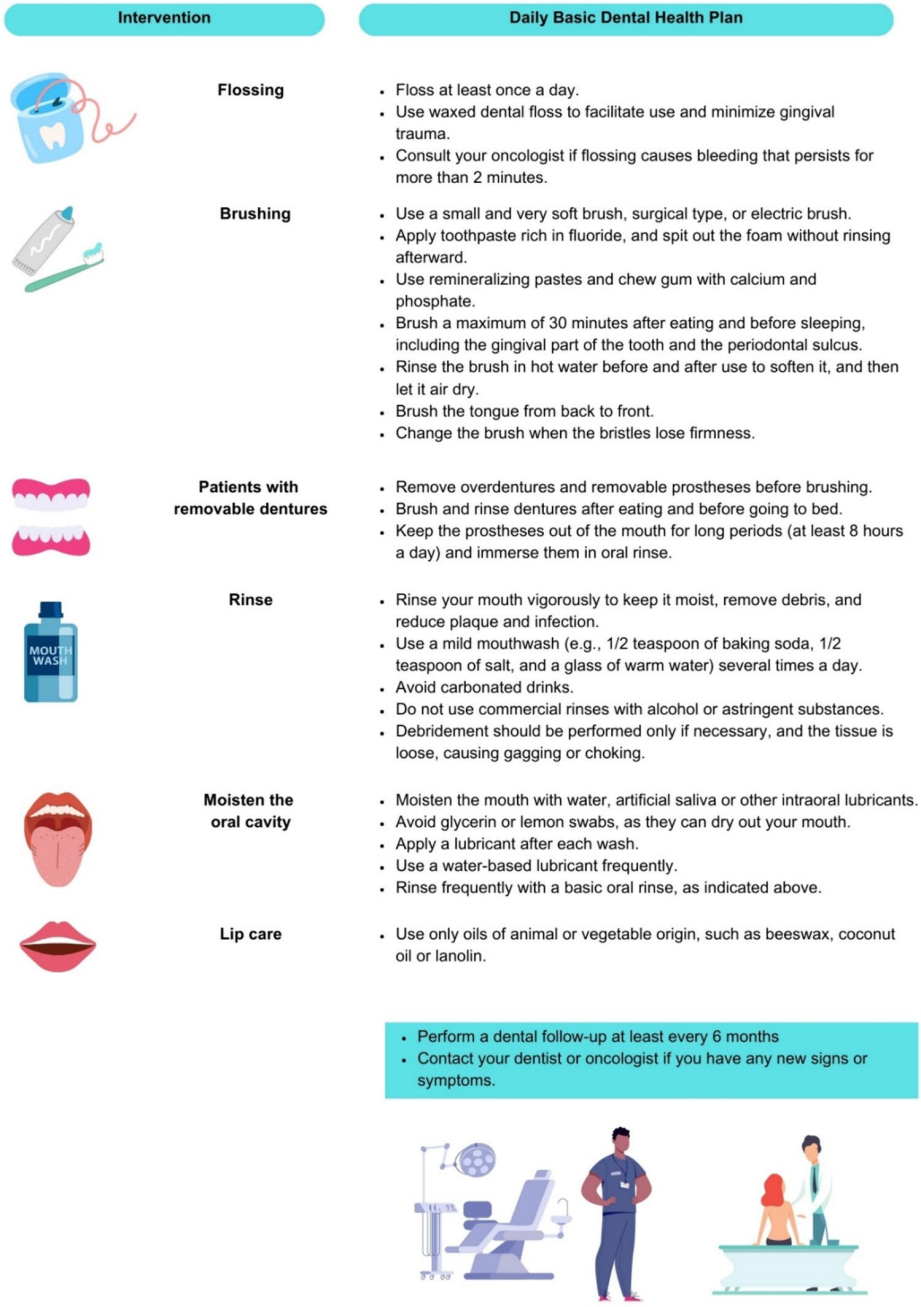
Dental care plan for patients with breast cancer

MRONJ is a complication in patients treated with drugs that affect bone biology. It is characterized by the exposure of necrotic bone in the mouth. The stages of MRONJ are shown in Table 3. It meets specific criteria, including treatment with bone biology-modifying agents and the absence of radiotherapy to the jaws [81]. Its prevalence is low (1–17%), but it is more common in patients with cancer than in those treated for osteoporosis [82, 83]. Risk factors included the use of specific drugs, chemotherapy, tumor type, concurrent corticosteroids, kidney disease, and demographic factors. Some medications still do not have sufficient evidence of their relationship with MRONJ, so recommendations must be personalized [84, 85]. The recommended dental care plan is shown in Fig. 6.

**Table 3:** Stages of osteonecrosis of the jaws due to medication

At risk	There is no exposed bone in patients being treated with drugs potentially causing the complication
Stage 1	Exposed bone or fistulas are present, but the patient is asymptomatic
Stage 2	Exposed bone and fistulas are associated with infection, pain, and erythema
Stage 3	Exposed bone and fistulas are associated with infection, pain, and erythema. In addition, one or more of the following signs are present: exposed bone beyond the alveolar parcel, pathological fracture, extraoral fistula, oroantral or oronasal communication, osteolysis that extends up to the mandibular basal or sinus floor

On the other hand, the toxicity associated with oncological treatments has a significant impact on the performance of invasive dental procedures and aesthetic treatments. These toxicities vary considerably between patients and are influenced by multiple factors, such as existing comorbidities, age, history of oncological treatments, and the immunodeficiency status of the individual [86]. Table 1 shows the risk of MRONJ and other toxicities associated with the most commonly used drugs for treating BC.

## Physiotherapy

Lymphedema, characterized by the accumulation of protein-rich fluid in the interstitial space due to lymphatic system dysfunctions, can progress to chronic inflammation, fibrosis, and vascular degeneration if left untreated It affects patients physically, psychologically, socially, and economically, impacting their self-esteem and body image. According to the International Society of Lymphology (ISL), the disease is divided into four stages: stage 0 (subclinical), stage I (spontaneously reversible), stage II (spontaneously irreversible) and stage III (elephantiasis) [87]. Lymphedema is often underestimated in oncology patients, and lymph node removal and radiation therapy are risk factors. Its appearance can be late, and it presents symptoms such as heaviness and alterations in bone contours [88–91]. Risk factors include lymph node removal, axillary radiation therapy, postoperative seromas, certain chemotherapy drugs, obesity, and hypertension [92, 93] with obesity and postoperative weight gain being especially significant factors for lymphedema of the lower extremities [94].

In the clinical setting, lymphedema secondary to oncological treatments commonly emerges between 12 and less than 24 months posttreatment, although its appearance can last up to a decade. The initial manifestations that appear in the upper limbs include a sensation of heaviness, alterations in the bone contours of the hand, and variations in volume, both proximal and distal. In the lower limbs, secondary lymphedema tends to originate near the trunk or genitals, less frequently in the feet, preceded by a feeling of heaviness before visible evidence of increased volume [89].

The education of health professionals for early diagnosis and effective treatment, as well as prospective surveillance, is crucial. Preventive strategies include regular monitoring and early physical therapy to reduce its incidence and improve patients’ quality of life [95, 96].

The early implementation of physical therapy in patients at high risk of lymphedema has proven effective in preventing this condition. Therapy encompasses comprehensive scar management, stretching, treatment of fascial retractions, progressive exercises, and education. Oncologists must recognize the importance of preventing and managing lymphedema from the early stages, with an accurate diagnosis and efficient intervention to delay its progression and improve patients’ quality of life [97–99].

Contrary to certain beliefs, activities such as blood drawing or air travel have not shown a direct association with lymphedema [99]. However, the individualized use of compression sleeves is recommended to prevent their appearance in high-risk patients [100, 101].

**Fig. 7:**
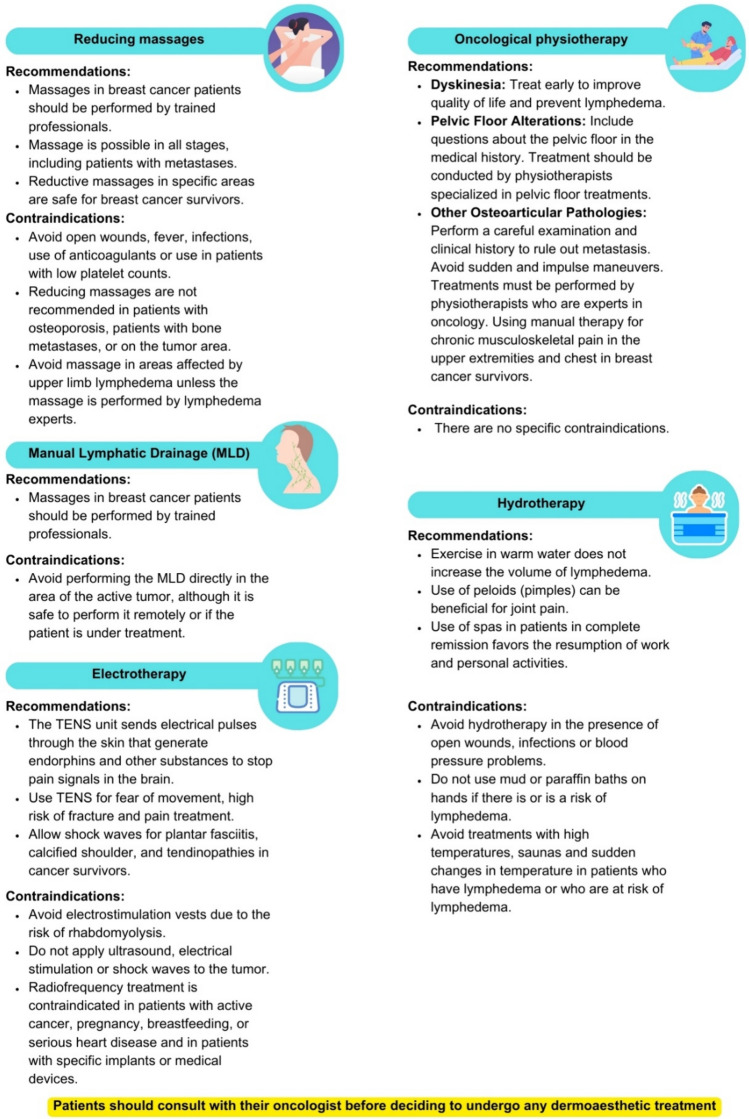
Recommendations and contraindications for physiotherapy treatments in patients with breast cancer

Recommendations and contraindications for the types of treatments that can be performed are shown in Fig. 7.

### Scar treatment

Several safe and effective physical therapy techniques, including myofascial induction, are used for the healing and management of scar disorders [102, 103]. This technique, which may include hooks, is efficacious in improving the appearance of the scar at superficial and deep levels and is especially useful in breaking up adhesions and restoring normal motion [103, 104].

Manual lymphatic drainage is adequate for the treatment of adhered areas after radiotherapy. At the same time, deep oscillation (DEEP OSCILATING© or HIVAMAT©) uses low-intensity electrostatic fields to relieve pain, reduce inflammation, and promote lymphatic drainage, benefiting cicatrization [105]. In addition, KINESIOTAPE© or a neuromuscular bandage, which is applied with variations in tension and direction, promotes healing by improving circulation [106, 107]. These techniques offer complementary approaches for effective scar management in physical therapy.

### Reducing massages

In oncology, “oncology massage” is a massage technique tailored to the needs of cancer patients that uses unique pressure and direction principles. It is beneficial for improving relaxation, sleep, and immune responses and relieving symptoms such as fatigue, pain, anxiety, and nausea [108–110]. This massage offers physiological benefits and essential physical contact, which is especially important in palliative care. Scientific evidence supports its safety in oncology patients and highlights its value as a supportive treatment in oncology [111, 112].

### Manual lymphatic drainage (MLD)

It is a gentle massage technique focused on the superficial lymphatic system that is safe and effective in BC patients [113, 114]. It does not increase the risk of recurrence or aggravate metastases [115]; it contributes to reducing lymphedema volume, improves joint mobility, increases the pain threshold, promotes venous flow, and reduces fatigue in treated patients [116–119].

### Oncological physiotherapy

This technique prevents and treats sequelae of cancer treatments, especially BC, and offers a comprehensive approach that includes gait analysis, posture, assessment of the shoulder girdle, and measurement of the affected arm [120, 121]. It incorporates techniques to treat dyskinesia, pelvic floor disorders, and other osteoarticular pathologies, improving quality of life and providing personalized recovery. Global Lymphatic Therapy (Godoy Method) and Complex Decongestive Therapy are highlighted for their effectiveness in normalizing lymphedema and optimizing treatment results [122, 123].

Furthermore, within physiotherapy, there are various techniques to treat dyskinesia, pelvic floor disorders, or other osteoarticular pathologies [124–126].

### Hydrotherapy

Medical hydrology, which explores the impact of mineral-medicinal waters on health, offers therapies such as spa or thermal cure, especially beneficial for BC patients [127]. These therapies improve quality of life, relieve pain, fatigue, and anxiety, and benefit aspects such as lymphedema and mobility [128, 129]. They also improve body image and treat dermatological problems such as dry skin [130]. Despite its observed benefits, more research is required to establish its definitive effectiveness and the appropriate protocols for each patient.

### Electrotherapy

Electrotherapy uses electric and electromagnetic fields to improve patient health, offering a variety of techniques from the most basic, such as transcutaneous stimulation, laser, ultrasound, and transcutaneous electrical nerve stimulation (TENS), to advanced procedures such as radiofrequency or diathermy. This therapeutic modality, which acts on sensory or motor fibers and produces vasomotor, chemical, or analgesic effects, requires an individualized approach to adapt to the specific needs of each patient [131].

## Nutrition

**Fig. 8:**
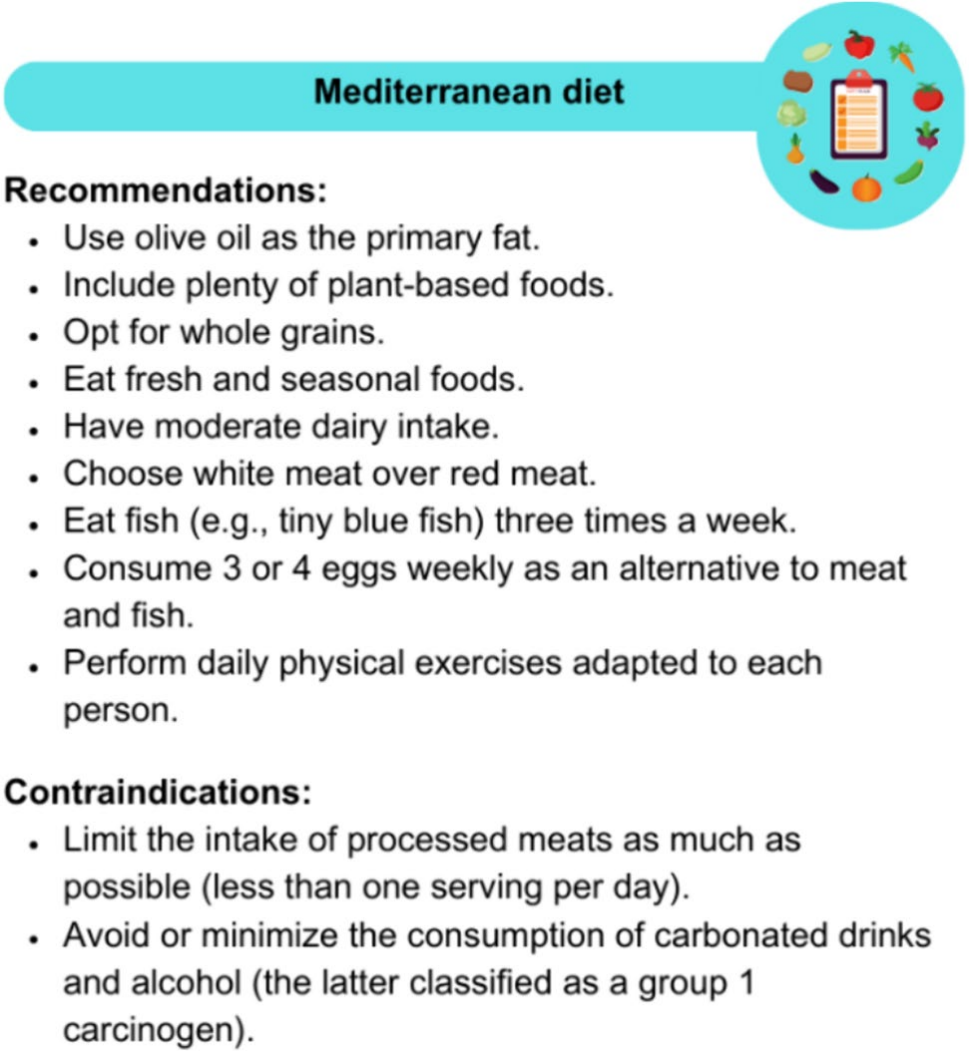
Recommendations and contraindications for following a Mediterranean diet

Effective weight management is essential for the prevention and treatment of BC, as many patients tend to gain weight after diagnosis [132]. Diets such as the Mediterranean diet, regular exercise, and stress management improve quality of life and reduce the risk of comorbidities [133–141]. These habits not only help prevent recurrences and improve the side effects of treatments in cancer patients but also increase the chances of survival [139, 140]. Nutrition focuses on dietary patterns and cultural aspects [142], highlighting the importance of consuming fruits and vegetables and engaging in physical activity [143]. This significantly reduces the risk of relapse and mortality, especially in BC patients [9, 144]. Figure 8 shows the guidelines for following a Mediterranean diet. Adherence to the Mediterranean diet can be assessed using a 14-item questionnaire, where a score of 9 or more indicates good adherence [145, 146].

On the other hand, alternative diets or the use of nutritional supplements are gaining popularity among BC patients to improve their quality of life. However, it is crucial to base these treatments on scientific evidence [147–149].

*Ketogenic diet* This dietary plan severely limits carbohydrates but has not yet been shown to affect cancer progression. However, it could have nutritional risks, and its usefulness in metastatic BC is being investigated [150].

*Intermittent fasting* This approach involves temporary restrictions on food intake. It could reduce the toxicity of oncological treatments, but more studies are required to establish clear guidelines [151].

*Alkaline diet* An alkaline diet seeks to alter the blood pH through diet, but there is no evidence that it influences cancer progression. Body pH is regulated by body systems and is not significantly altered by diet [152].

*Metabolic cancer therapy* This treatment uses artificial diets that modify amino acids and restrict lipids. It has shown promising results in mouse studies, especially in triple-negative BC, but more research is needed [153].

*Natural supplements* These supplements can interact with oncological treatments and influence their effectiveness [154]. Due to possible interactions, the American Society of Integrative Oncology does not recommend the use of natural supplements in cancer patients [155].

## Gynecology

**Fig. 9:**
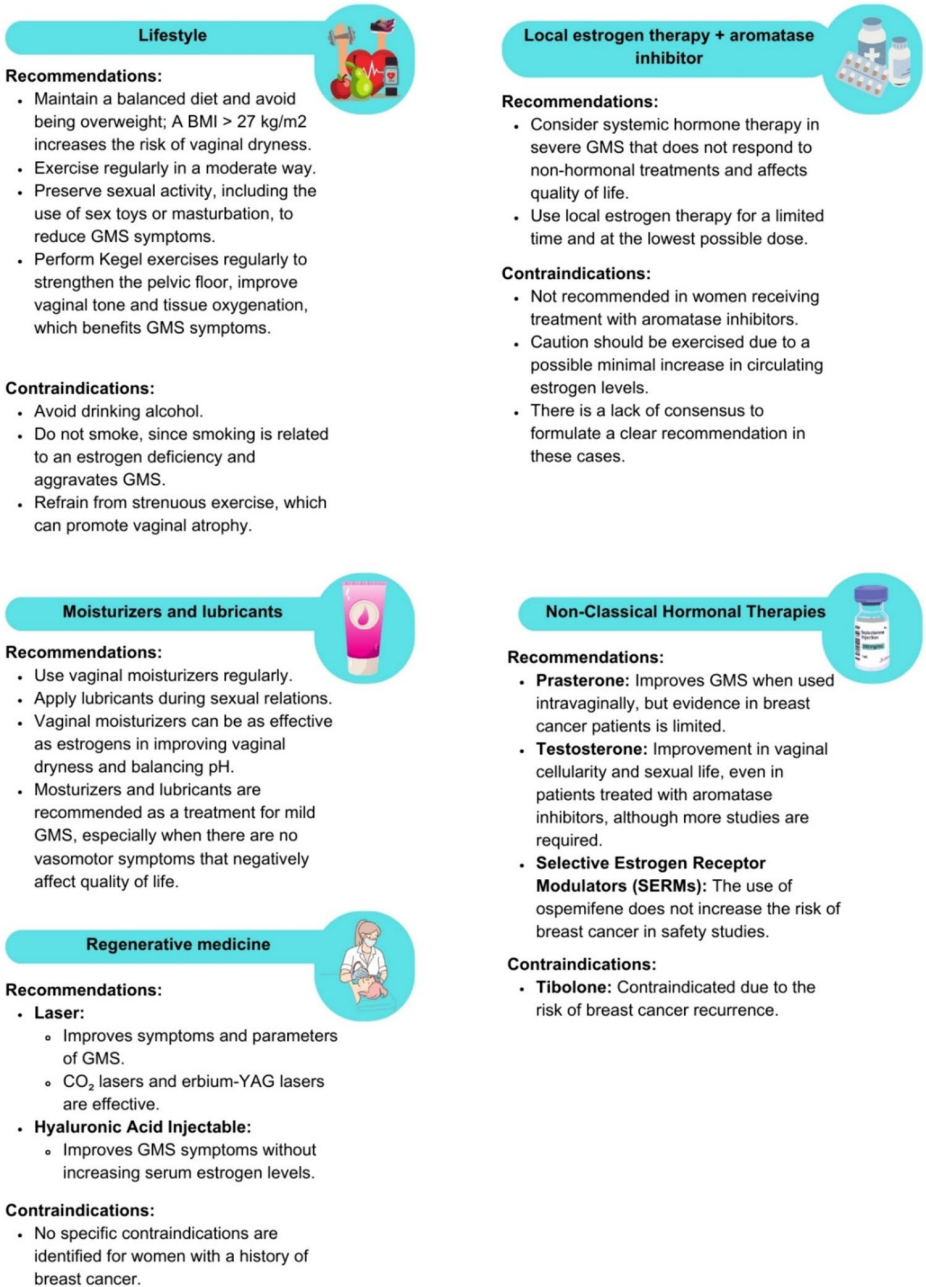
Recommendations and contraindications for treatments aimed at improving genitourinary syndrome during menopause that can be performed by patients with breast cancer

In the comprehensive treatment of patients with BC, it is crucial to address aspects that impact their quality of life, including sexual health and side effects derived from treatments. Sexual activity can be beneficial, contributing to improving local vascularization and relieving symptoms of GMS [156, 157]. The genitourinary changes in GMS and their clinical impact are shown in Table 4. The recommendations and contraindications for treatments aimed at improving GMS symptoms are shown in Fig. 9.

**Table 4:** Genitourinary changes in the genitourinary syndrome of menopause and its clinical impact

Genitourinary changes	Signs and symptoms
Loss of thickness and elasticity of the epithelium	Discomfort, burning, itching. Presence of ecchymosis, ulcers with trauma (intercourse or gynecological examination)
Increase in subepithelial connective tissue. Loss of roughness. Shortening and lack of compliance	Dyspareunia
Reduction of secretion and transudate production	Dryness. Dyspareunia. Decreased sexual desire
Increase in vaginal pH	Increased predisposition to infections
Vulvar alterations	Anatomical changes. Loss of self-esteem. Decreased sexual satisfaction
Decreased thickness of urethral epithelium and trigone	Dysuria, frequency, nocturia, urinary urgency, increased frequency of urinary tract infection
Pelvic floor muscles, endopelvic fascia	Increased tendency to pelvic organ prolapse. Stress incontinence

### Lifestyle

Adopting a healthy lifestyle is essential not only for general health but also for genitourinary health. Therefore, specific measures to improve lifestyle during menopause, which can have a positive impact on the well-being of BC patients, are recommended [158, 159].

### Use of vaginal lubricants and moisturizers

Vaginal lubricants and moisturizers are options for use in GMS, especially when hormonal or pharmacological treatments are contraindicated [160]. They relieve the symptoms of GMS and can be used in conjunction with other treatments. The differences between lubricants and moisturizers can be found in Table 5.

**Table 5:** Differences between lubricants and moisturizers

Domain	Lubricants	Moisturizers
Description and function	They resemble natural vaginal secretions	They are absorbed and adhere to the vaginal lining, simulating natural secretions. They reduce vaginal pH, maintaining the humidity and acidity of the vagina
	Discomfort due to vaginal dryness, especially if it only occurs during intercourse	Discomfort due to vaginal dryness during sexual intercourse or on a daily basis, even in women who are not sexually active
Application and long term effects	They are applied to the vagina, vulva and, if necessary, the partner’s penis before sex They are also necessary in the use of sex toys or vaginal dilators	They are applied regularly once every 1–3 days, depending on the intensity of vaginal dryness
	Its action is fast, but for a limited time Water-based lubricants reduce genital symptoms to a greater extent than silicone-based lubricants In addition, they do not stain and seem to be associated with a better sexual response The use of oil or silicone-based lubricants is discouraged	Its effect lasts 2 to 3 days Most contain water, as well as synthetic or plant-based polymers and other excipients to ensure appropriate pH, osmolarity and viscosity. Moisturizers with hyaluronic acid are especially beneficial, as they have proven effective against placebo
Safety and composition considerations	The pH should be between 3.8 and 4.5, similar to the vaginal pH. On the other hand, the osmolarity of a lubricant should be less than 380 mOsm/kg to minimize epithelial damage Some lubricants contain parabens, which could act as endocrine disruptors	The pH must be similar to the vaginal one and the osmolarity low, if possible lower than the threshold of 380 mOsm/kg recommended by the WHO

### Local estrogen therapy (LET)

LET is a therapeutic option for GMS, especially in nonhormone-dependent BC patients. Therapy focuses on relieving GMS symptoms through the local application of estrogen.

Current evidence supports the use of local estrogens in nonhormone-dependent BC survivors, especially for hormone receptor-negative tumors, which are common in young women. In these patients, GMS symptoms are less prevalent in the first few years. On the other hand, in patients with hormone-dependent tumors treated with aromatase inhibitors, some studies have indicated a slight increase in estradiol in the blood, but the results are contradictory [161, 162]

Evidence shows that the use of LET in patients treated with tamoxifen is not associated with worse overall or disease-free survival outcomes. The most suitable option would be estriol because it cannot be transformed into estradiol or estrone, and very low-dose formulations are available (Table 6).

**Table 6:** Local estrogens

Composition	Product	Dosage
Promestriene 10 mg/application	COLPOTROFIN^®^ 1% vaginal cream (LABORATOIRES CHEMINEAU)	**Start:** 1 application every day, preferably at night
		**Maintenance:** 2/week
Estriol 1 mg/g	OVESTINON^®^ vaginal cream 0.1% (UNITHER INDUSTRIES)	**Start:** 1 application every day, for 2 weeks
		**Maintenance:** 0.5 mg every 2 weeks
Estriol 1 mcg/g	BLISSEL^®^ vaginal gel 50 mcg/g (ITALFARMACO S.A)	**Start:** 1 application every day, for 2 weeks
		**Maintenance:** 2 times a week
Estradiol 10 mcg/application	VAGIFEM^®^ coated vaginal tablet 10mcg (NOVO NORDISK A/S)	**Start:** 10 mg/day for 2 weeks
Mucoadhesive tablet		**Maintenance:** 10 mg/day 3 times a week
Estradiol 10 µg tablet	**VAGIRUX**^**®**^ (GEDEON RICHTER PLC)	**Start:** 10 mg/day for 2 weeks
		**Maintenance:** 20 mg 2 times a week
Estradiol silastic ring	**ESTRING**^**®**^ (QPHARMA AB)	7.5 mg/day

### Regenerative medicine

Laser treatment and the use of injectable hyaluronic acid are innovative techniques for addressing symptoms such as vaginal atrophy and dryness associated with GMS. Laser technology, recently introduced as a therapeutic alternative [163], and injectable hyaluronic acid, although mainly documented in case series without a control group, are promising since hyaluronic acid does not increase serum estrogen levels [164, 165].

### Hormone therapy

#### Oral and systemic hormone therapy

Although hormone therapy is primarily used for the management of general menopausal symptoms such as hot flashes and mood swings, it may also have effects on GMS symptoms. Menopausal hormone therapy (MHT) may influence genitourinary health, especially vaginal dryness and other vaginal and urinary symptoms associated with menopause [166].

Table 7 shows a detailed outline of the safety and recommendations of different hormonal treatment modalities, classified as combination therapy, estrogen-only therapy, and tibolone treatment. This classification is based on the evaluation of specific risk factors associated with different patient profiles, from breast cancer survivors with hormone receptor-positive or negative tumors to carriers of the BRCA1 and BRCA2 genetic mutations.

**Table 7:** Menopausal hormone therapy and breast cancer eligibility criteria

Condition		Combined MTH		Estrogen-only MTH		Tibolone	Clarifications
		Oral	Trsd	Oral	Trsd		
Breast cancer	Breast cancer survivors with RH (−) tumors	2	2	2	2	3	
	Breast cancer survivors with HR (+) tumors	3	3	3	3	4	Expert opinion
	BRCA1 carrier	2	2	2	2	NA	
	BRCA2 carrier	2	2	2	2	NA	Extrapolation of BRCA1 (expert opinion)

Category 1: No restrictions on the use of MHT; Category 2: The benefits outweigh the risks; Category 3: Risks generally outweigh benefits; Category 4. MHT should not be used; NA: Not applicable due to lack of available evidence

#### Nonclassical hormone therapy

In the context of BC management, it is crucial to consider nonclassical hormonal therapeutic alternatives, such as prasterone, testosterone, and selective estrogen receptor modulators (SERMs), as they can be used to improve GMS symptoms [167–170].

## Conclusions

This review provides practical and applicable recommendations for the comprehensive treatment of patients with BC. This highlights the relevance of a multidisciplinary approach that goes beyond the mere eradication of cancer, which positively affects patients' physical and psychological recovery. In this context, patients should take the necessary precautions and consult their oncologist before undergoing dermoaesthetic treatment. Implementing these practices in clinical care enhances their usefulness in significantly improving patients' quality of life, underscoring the effectiveness of personalized and well-coordinated treatment between various medical specialties.

## Data Availability

Not applicable.
